# Quality of life and quality of education among physiotherapy students in Europe

**DOI:** 10.3389/fmed.2024.1344028

**Published:** 2024-02-19

**Authors:** Michaela Schramlová, Kamila Řasová, Johanna Jonsdottir, Markéta Pavlíková, Jolana Rambousková, Marja Äijö, Martina Šlachtová, Alena Kobesová, Elena Žiaková, Turhan Kahraman, Dagmar Pavlů, Beatriz María Bermejo-Gil, Daphne Bakalidou, Evdokia Billis, Papagiannis Georgios, José Alves-Guerreiro, Nikolaos Strimpakos, Aleš Příhoda, Marika Kiviluoma-Ylitalo, Marja-Leena Lähteenmäki, Jana Koišová, Gentiana Berisha, Magdalena Hagovská, Anna Laura Arca, Sara Cortés-Amador

**Affiliations:** ^1^Department of Rehabilitation, Third Faculty of Medicine, Charles University, Prague, Czechia; ^2^IRCCS Fondazione Don Carlo Gnocchi ONLUS, Milan, Italy; ^3^Department of Hygiene, Third Faculty of Medicine, Charles University, Prague, Czechia; ^4^Savonia University of Applied Sciences School of Health Care, Kuopio, Finland; ^5^Department of Physiotherapy, Faculty of Physical Culture, Palacky University, Olomouc, Czechia; ^6^Department of Rehabilitation and Sports Medicine, Second Faculty of Medicine, Charles University and University Hospital Motol, Prague, Czechia; ^7^Department of Physiotherapy, Faculty Nursing and Professional Health Studies, Slovak Medical University in Bratislava, Bratislava, Slovakia; ^8^Department of Health Professions, Faculty of Health and Education, Manchester Metropolitan University, Manchester, United Kingdom; ^9^Faculty of Physical Education and Sport, Charles University, Prague, Czechia; ^10^Department of Nursery and Physiotherapy, Faculty of Nursery and Physiotherapy, Universidad de Salamanca, Salamanca, Spain; ^11^Laboratory of Neuromuscular and Cardiovascular Study of Motion (Lanecasm), Department of Physiotherapy, University of West Attica, Egaleo, Greece; ^12^Department of Physiotherapy School of Health Rehabilitation Sciences, University of Patras, Aigio, Greece; ^13^Biomechanics Laboratory, Physiotherapy Department, University of the Peloponnese, Sparta, Greece; ^14^Center for Innovative Care and Health Technology (ciTechCare), School of Health Sciences (ESSLei) Polytechnic of Leiria, Leiria, Portugal; ^15^Health Assessment and Quality of Life Lab Department of Physiotherapy, University of Thessaly, Volos, Greece; ^16^Division of Musculoskeletal & Dermatological Sciences, University of Manchester, Manchester, United Kingdom; ^17^Department of Health Care Disciplines and Population Protection, Faculty of Biomedical Engineering, Czech Technical University in Prague, Prague, Czechia; ^18^SAMK – Satakunta University of Applied Sciences, Pori, Finland; ^19^Tampere University of Applied Sciences, Tampere, Finland; ^20^Faculty of Health Sciences, University of Ss. Cyril and Methodius in Trnava, Trnava, Slovakia; ^21^Universum International College Pristina, Pristina, Kosovo; ^22^Department of Physiatry, Balneology, and Medical Rehabilitation, Faculty of Medicine, PJ Safarik University, Kosice, Slovakia; ^23^Coordinator of Physiotherapist School Traineeship AOU, Sassari, Italy; ^24^Physiotherapy in Motion, Multispecialty Research Group (PTinMOTION), Department of Physiotherapy, Faculty of Physiotherapy, University of Valencia Gascó Oliag n Valencia, Valencia, Spain

**Keywords:** students, physiotherapy, stress, nutrition, sleep, physical activity

## Abstract

**Background:**

The study of physiotherapy is challenging and can affect the students’ well-being and quality of life. The aim of this study was to describe and compare factors that could affect well-being among students across Europe.

**Methods:**

In this descriptive cross-sectional study using an online questionnaire survey, students of bachelor’s physiotherapy programs from 23 European faculties, from 8 countries, were interviewed on mental health and stress burden, sleep quality, dietary habits, and physical activity.

**Results:**

Although 75% of students rated their quality of life positively and 47% were satisfied with their mental health, 65% showed higher levels of stress and 51% described impaired sleep quality. The minimum physical activity of 150 min weekly was described by 79% of students, within which 67% engaged in strengthening twice a week. Students with a higher stress load/worse psychological health also showed worse sleep quality and lower amount of physical activity, women were significantly worse off. In terms of physical activity and sleep quality, students from Finland and Kosovo achieved the best results, while students from Italy, Greece, and Portugal achieved the worst. Students from Italy indicated the greatest dissatisfaction with the organisation of the study system and communication with teachers, while in Kosovo students rated the communication and study organisation the highest. All students had a problem with adhering to nutritional habits. Students from Italy and Spain, with the lowest body mass indexes and weight averages, were closest to the nutrition recommendations.

**Conclusion:**

We demonstrated that physiotherapy students are burdened with stress, suffer from sleep disorders, and do not follow the recommendations regarding nutrition nor physical activity. There are significant differences between universities and countries in some aspects.

## 1 Introduction

Physiotherapy education varies worldwide, with some countries offering on-the-job training while others have bachelor’s or master’s degree programs. There are also differences in postgraduate education across Europe (1). Teaching techniques to future physiotherapists also present challenges due to variations in learning styles and attitudes towards clinical-practical teaching. National universities and their faculties can differ in various ways, and health systems and policies impact rehabilitation and physiotherapy methods, too. There is a limited number of empirical studies comparing the experience of physiotherapy students at different institutions (2–4), highlighting the variations in physiotherapy education worldwide (5, 6). In this study, we focused on the comparison of bachelor’s degree programs in physiotherapy in Europe.

During the bachelor’s study program, students learn, from a physiotherapeutic point of view, within the framework of complex rehabilitation treatment, to take an anamnesis, establish a differential diagnosis and prognosis (e.g., based on kinesiological analysis, examination of functional disorders of the musculoskeletal system, examination of clinical functions according to standardised and validated tests), design a short-term and long-term therapeutic plan and carry out effective therapy (e.g., treatment of functional disorders of the locomotor system, mobility, spasticity, pain, fatigue, improvement of physical and psychological condition, and quality of life). It is challenging because it requires: (1) extensive study of theoretical knowledge in preclinical and clinical fields of medicine, (2) development of manual, communicative and empathic skills in subjects specialised in acquiring professional expertise, and (3) understanding of scientific work in subjects focused on the preparation of a bachelor’s thesis. To sum it up, such education needs good health and mental condition of the students.

Recently, great emphasis has been placed on well-being that encompasses the quality of life and the ability of people and societies to contribute to the world with a sense of meaning and purpose (7). Students’ well-being could be influenced by many factors, e.g., physical and mental conditions, educational attainment, occupational status, leisure activities, leisure time, social affiliation, religious security, physical security or personal autonomy (8), university access, rigorous curricula, clinical practice obligations, financial pressures (9, 10), sleep patterns, diet, and physical activity (11), the impact of the pandemic or university background and study conditions (12). There were some studies presented by colleagues (13–15) that dealt with students’ quality of life. However, these were mixed student populations or students from only one country.

That is why we carried out this descriptive cross-sectional study using an online questionnaire survey with the aim to describe and compare the current educational systems and physical and mental well-being of physiotherapy students in Europe. Physical and mental well-being were divided into the following subcategories: mental health and associated stress levels (2, 4, 16, 17), sleep quality and patterns (18, 19), dietary habits (20, 21), and physical activity levels (22, 23). These subcategories were analysed in relation to each other, highlighting their interconnectedness and importance within the broader framework of the quality of life (24–27).

## 2 Method

### 2.1 Description of the project

The overall project consisted of two phases. In the first phase, carried out in 2021, two survey questionnaires were developed. The first questionnaire aimed to systematically describe organisational aspects of physiotherapy faculties by their representatives. The second questionnaire focused on describing the physiotherapy students’ well-being. Moreover, a list of potential participating universities was prepared and the approvals of the ethics committees of participating faculties were obtained. In the second phase, carried out between February and December 2022, the data were collected.

### 2.2 Study design

A descriptive, cross-sectional online survey, using self-administered online questionnaires.

### 2.3 The survey questionnaire

The lead author (MS) developed an initial draft of the questionnaire that was agreed upon in five rounds of core group (MS, KŘ, MP, MA, JJ, JR) email communication. It was piloted with 60 students from three European universities in 2021. Based on pre-analyses, the core group agreed on the final questionnaire items and wording. Then, the internet version using the SURVIO.cz portal was developed.

#### 2.3.1 The questionnaire for guarantors

The questionnaire for guarantors comprised 16 questions concerning organisational aspects of participating universities’ programs: how many students undergo the bachelor program, what type of study program is offered (bachelor, master, doctoral), how many semesters students’ study to reach bachelor’s degree, what is the form of study (present, distant or combined), should students pay for the study, whether there is a possibility to reach scholarships, etc.

#### 2.3.2 The questionnaire for students

The questionnaire for physiotherapy students consisted of 87 questions divided into three parts (Supplement 1).

The first part collected background information, such as gender, age, weight and height, university and semester of study, and subjective level of English.

The second part focused on the students’ quality of life, covering:

##### 2.3.2.1 Stress and mental health

These were analysed using the Undergraduate Sources of Stress questionnaire, USOS and the World Health Organization Quality of Life Questionnaire – short version, WHOQOL-BREF. USOS is a questionnaire specifically aimed at evaluating the degree of stress load among university students, evaluating 3 categories of potential stressors (academic, financial, and personal). The maximum number of points could be 72, which would be interpreted as maximally stressful.

Six questions were selected from the WHOQOL-BREF questionnaire assessing various aspects of quality of life, including overall life quality, mental health satisfaction, enjoyment of life, perception of life meaning, self-satisfaction, and sense of control. The maximum number of points could be 24, which would be interpreted as the worst subjective perception of quality of life.

##### 2.3.2.2 Sleep quality

It was analysed based on the Pittsburgh Sleep Quality Index (28) and questions combining WHOQOL-BREF and the study (18). Pittsburgh Sleep Quality Index, PSQI, measures several different aspects of sleep as sleep quality, sleep latency, sleep duration, habitual sleep efficiency, sleep disturbances, use of sleeping medication, and daytime dysfunction. The maximum number of points could be 21, which would be interpreted as the worst quality of sleep. Impaired sleep quality is indicated by a score of 5 or more (29).

##### 2.3.2.3 Dietary habits

We were interested in sufficient intake of fluids, fruit, vegetables, and alcohol intake per day (questions were formulated based on WHO recommendations (30, 31)). Nutritional habits were reflected in body weight, and therefore we assessed Body Mass Index (BMI). Moreover, the importance and satisfaction with current nutrition education were questioned.

##### 2.3.2.4 Physical activity

Questions concerning physical activity were formulated based on the International Physical Activity Questionnaire – Short Form and the Food & Physical Activity Questionnaire (32) (in terms of the amount and duration of strengthening and relaxation/meditation exercises and the number of steps) and quantified using METs recommended by WHO (33). WHO recommends at least 150–300 min of moderate-intensity aerobic physical activity or at least 75–150 min of vigorous-intensity aerobic physical activity.

##### 2.3.2.5 Employment

The third part was devoted to the satisfaction with the university background and study conditions.

### 2.4 Recruitment process

A total of 45 European universities were identified by the core team. Of these, representatives from 30 faculties confirmed their participation. They were regularly contacted every month to optimise the survey response rate. Seven of the faculties were excluded because they did not provide sufficient responses (0–1 response from students). Finally, 23 faculties from 20 universities from 8 countries participated. Representatives from each faculty co-ordinated the data collection individually – it was recommended to organise a lecture explaining the importance to participate and advertise to fill the questionnaire regularly.

### 2.5 Inclusion criteria

The first questionnaire was filled out by specialists in physiotherapy (guarantors of the study programmes or teachers at universities, who knew general information about the university and physiotherapy study programmes).

The criteria for respondents of the second questionnaire were: (a) a full-time student of physiotherapy, in the bachelor’s study program, in the academic year 2021/2022 or 2022/2023; (b) demonstrating sufficient English language proficiency to comprehend the survey questions.

### 2.6 Data analysis

The data from the first questionnaire was processed to create an overview presented in Table 1. The data from the second questionnaire were analysed for the whole sample as well as separately for each of the countries. In this article, only the data comparing individual countries are presented; data in individual universities are mentioned only if notable differences occurred.

**Table 1:** Participating countries and universities, basic characteristics.

Country	Faculty, University, Town	Higher study levels		BSc. study program		Financial aspects		Number of students		
		MSc.	PhD.	Semesters	Form	Student fees	Scholarship available	Replied	Total number of PT students/addressed	Response rate
Czech republic	2nd Medical Faculty, Charles University, Prague	✓	✓	6	Present	×	✓	25	60	41.7%
	3rd Medical Faculty, Charles University, Prague	×	✓	6	Present	×	✓	67	88	76.1%
	Faculty of Physical Education and Sport, Charles University, Prague	✓	×	6	Present	×	✓	12	150	8.0%
	Faculty of Physical Culture, Palacký University Olomouc	✓	×	6	Present	×	✓	51	93	54.8%
	Faculty of Biomedical Engineering, Czech Technical University in Kladno	✓	×	6	Present	×	✓	47	102	46.1%
Finland	Savonia University of Applied Sciences, Kuopio	×	×	7	Present	×	×	59	141	41.8%
	Satakunta University of Applied Sciences, Satakunta	×	×	7	Present	×	×	13	75	17.3%
	Tampere University of Applied Sciences, Tampere	×	×	7	Present	×	×	13	120	10.8%
	Oulu University of Applied Sciences, Oulu	×	×	7	Present	×	×	10	NA	–
Greece	University of Peloponnese	×	✓	8	Present	×	×	134	336	39.9%
	University of West Attica	✓	✓	6	Present	×	×	198	NA	–
	University of Thessaly	✓	✓	8	Present	×	×	46	465	9.9%
	University of Patras	✓	✓	8	Present	×	×	119	460	25.9%
Italy	University of Milan	✓	×	6	Present	✓	×	7	83	8.4%
	University of Sassari	×	×	6	Present	✓	✓	11	NA	–
Kosovo	Universum College, Pristina	×	×	6	Present	✓	✓	22	NA	–
Portugal	Politécnico de Leiria, Leiria	×	×	8	Present	✓	×	114	221	51.6%
Slovakia	Pavol Jozef Šafárik University in Košice	✓	✓	6	Combined	×	✓	18	45	40.0%
	University of Ss. Cyril and Methodius in Trnava	×	✓	6	Combined	✓	✓	36	510	7.1%
	Slovak Medical University in Bratislava	✓	×	6	Combined	×	×	41	70	58.6%
Spain	University of Salamanca, Salamanca	×	✓	8	Present	✓	✓	16	195	8.2%
	University of Valencia, Valencia	✓	✓	8	Present	✓	✓	25	NA	–
Total								1,084	3,214*	33.7%*

NA, not available (not provided by the university). *Total number includes available information only. Real response rate may be thus slightly lower.

Continuous variables were summarised using the mean with standard deviation (SD) and/or the median with interquartile range (IQR). Absolute and/or relative frequencies were used to summarise categorical variables. Differences between groups (women vs. men, countries) were compared using *χ*^2^-test in the case of categorical variables or the *t*-test/ANOVA *F*-test in the case of continuous variables. Pearson’s correlation coefficient (*r*) was used to assess the relationship between various continuous or five-level ordered variables concerning stress and sleep quality. Similarly, *χ*^2^-test was used to assess relationship between categorical variables. The level of statistical significance was set at the 0.05 level. The statistical environment and language used for analysing was R, version 4.0.2 (23).

## 3 Results

### 3.1 Organisation of physiotherapy studies across Europe

Management of 23 European faculties was described. Apart from bachelor’s study program, 12 faculties offer also master and 11 faculties doctoral programs. Finnish universities offer a 7-semester undergraduate degree programme, while Greek, Portugal and Spanish an 8-semester. The remaining universities follow the standard 6-semester format. The largest universities by student enrolment are the University of Trnava in Slovakia (having a total of 510 students across all 3 years) and the University of Patras in Greece (having 460–540 students in all years). The smallest university is the University of Košice in Slovakia, with only 45 students for all three years. A combined form of study is available exclusively at universities in Slovakia. Tuition fees are required at six universities (Kosovo, Salamanca, Trnava, Milan, Sassari and Leiria) with considerable variation both between and within institutions (Table 1).

### 3.2 Well-being of physiotherapy students

Out of the 3,214 students who were contacted by representatives from each participating universities, 1,084 responded, resulting in a response rate of 33.7%. Nine of them were students of the master program, so they were excluded. Data from 1,075 students were analysed. 67.8% of respondents were women with an average age 21.8 ± 4.6 years (the youngest students are from Kosovo, while the oldest from Finland) (Table 2).

**Table 2:** Demographic and baseline characteristics of participants.

Country	Number	Gender (%)		Age [years] mean (SD)	Weight [kg] mean (SD)	Height [cm] mean (SD)	BMI mean (SD)
		Female	Male				
Czech Republic	202	80.2%	19.8%	21.3 (2.1)	66.7 (12.6)	171.8 (9)	22.5 (3.2)
Finland	95	76.3%	23.7%	25.7 (6.3)	69.6 (13.6)	169 (8.3)	24.3 (3.9)
Greece	492	57.3%	42.7%	21.3 (4.7)	68.7 (13.6)	171.8 (9.6)	23.2 (3.6)
Italy	16	56.2%	43.8%	23.6 (4.4)	62.7 (13)	170.2 (10.3)	21.4 (2.7)
Kosovo	22	63.6%	36.4%	19.8 (1.3)	70.5 (14.3)	173.6 (9.2)	23.2 (3.1)
Portugal	114	71.9%	28.1%	21.4 (4.1)	63.9 (11.6)	165.8 (8.4)	23.2 (3.5)
Slovakia	93	78.5%	21.5%	23.3 (5.8)	66.6 (16)	170.4 (9.5)	22.9 (5.1)
Spain	41	82.9%	17.1%	20.6 (2.5)	62.9 (10.2)	167.6 (7.4)	22.3 (2.7)
TOTAL	1,075	67.8%	32.2%	21.8 (4.6)	67.5 (13.4)	170.6 (9.4)	23.1 (3.7)

#### 3.2.1 Stress and mental health

On average, the students achieved a score of 27.0 ± 10.6 points on the USOS questionnaire, with statistically significantly higher scores recorded by women compared to men (27.7 vs. 25.6, *p* = 0.005). Furthermore, 56.6% of the students referred to were experiencing high levels of distress. It is worthy to note that this condition was observed more often amongst women (*p* = 0.01) and those from Italy (94%, *p* < 0.001), but less frequently in students from Finland. Although over 50% of the students reported high stress levels, 75.3% objectively evaluated their quality of life as “good” or “very good”, and 47% reported being “satisfied” or “very satisfied” with their mental state (Table 3).

**Table 3:** Summary of stress, sleep, nutrition, physical activity, and employment across countries.

Country	USOS [range 0–72]	WHOQOL-BREF [range 0–24]	PSQI scores [range 0–21]	PSQI 5+	Liquid intakes [at least 1.5 L/day]	Alcohol intakes [at least 1 drink/week]	Physical activity [METs min/week]	HEPA	Paid employment
	Mean (SD)	Mean (SD)	Mean (SD)	%	%	%	Mean (SD)	%	%
Czech Republic	25.9 (8.9)	8.9 (3.9)	6.2 (3.0)	68%	73%	75%	2,118 (1439)	31%	49%
Finland	21.5 (8.7)	7.5 (4.1)	5.4 (2.6)	55%	75%	58%	2,152 (1362)	41%	41%
Greece	28.0 (11)	8.7 (4.1)	7.0 (3.4)	75%	73%	71%	1821 (1413)	29%	26%
Italy	31.8 (8.3)	10.7 (5.1)	6.1 (2.6)	69%	81%	81%	1791 (1180)	31%	25%
Kosovo	23.4 (13.6)	5.6 (4.5)	6.7 (3.6)	64%	86%	23%	1,668 (1603)	27%	41%
Portugal	29.9 (11.4)	8.6 (4.0)	6.9 (3.0)	75%	54%	55%	1,446 (1615)	23%	16%
Slovakia	25.2 (9.6)	7.5 (4.0)	6.4 (3.2)	69%	76%	58%	1994 (1519)	31%	58%
Spain	28.5 (10.0)	8.0 (3.3)	6.0 (3.0)	63%	68%	66%	2,160 (1446)	39%	17%
total	27.0 (10.6)	8.5 (4.1)	6.6 (3.2)	70.9%	71.5%	66.8%	1891 (1458)	30.5%	33.4%
Comparison between countries	*p* < 0.001*	*p* < 0.001*	*p* < 0.001*	*p* = 0.004^#^	*p* = 0.003^#^	*p* < 0.001^#^	*p* = 0.002*	*p* < 0.001^#^	*p* < 0.001^#^

*Differences between countries assessed using *F*-test in ANOVA model. #Differences between countries assessed using Pearson’s *χ*^2^-test.

**Figure 1:**
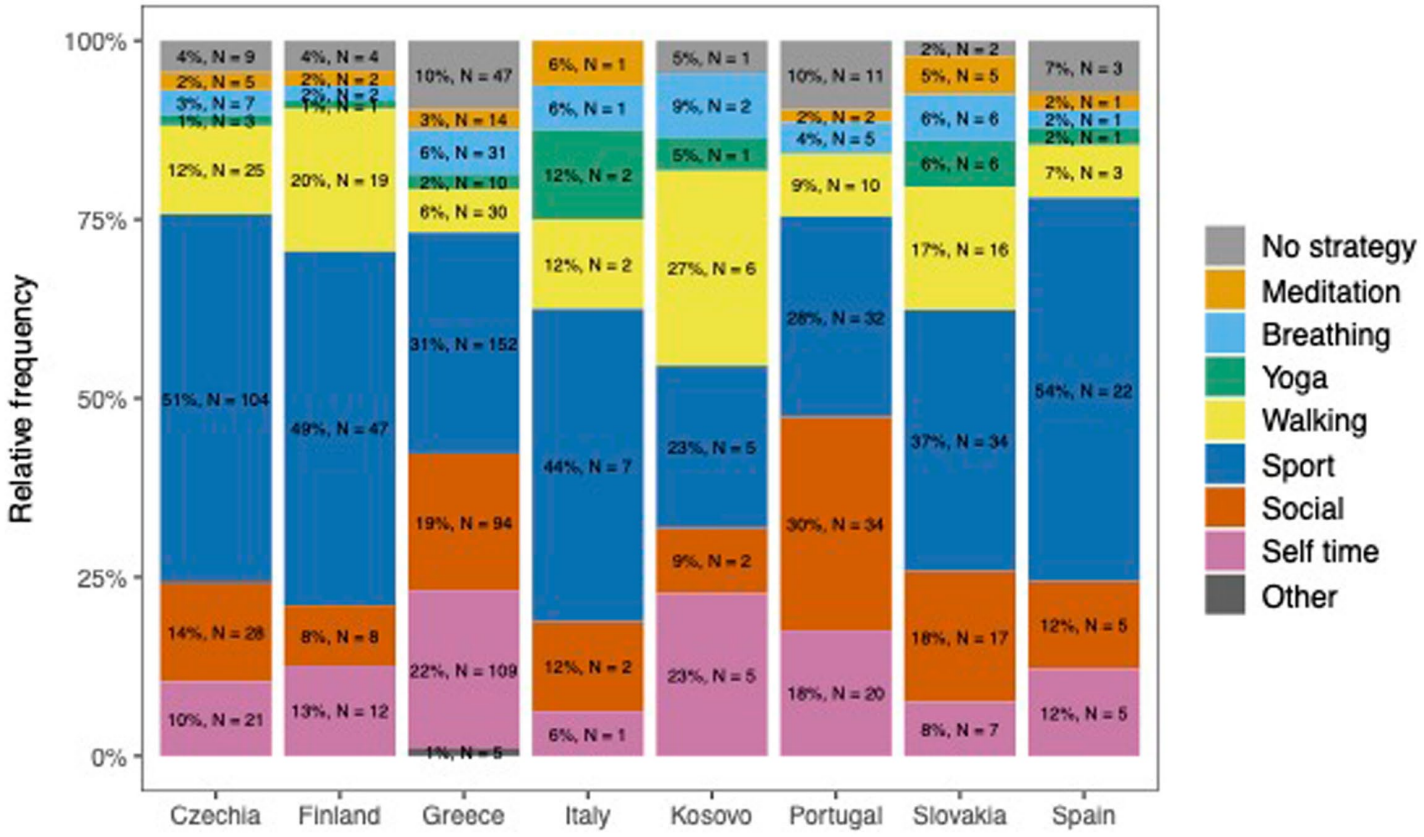
Students coping strategies among states.

Only 37.5% of students used physical exercise as a means of coping with high levels of stress, with a higher incidence among men compared to women (47.4% versus 32.7%), while only 5% of students practised yoga, breathing exercises, or meditation as a coping strategy, with a higher incidence among women (10.9% versus 8.4%). Other coping mechanisms included spending time with family or friends (17.7% of students), spending time in solitude (16.7%), and going for walks (10.3%). Only two students reported seeking professional help from a psychologist or psychotherapist. Three students mentioned smoking and drinking alcohol as coping mechanisms, while the majority did not use any specific strategy. Significant findings indicate that compared to other countries, students in Kosovo do not use physical activity as an important coping strategy (*p* < 0.001), but they do use walking and spending time alone. Italy had the highest usage of yoga as a coping strategy, while Portugal and Greece had the highest number of students reporting no coping mechanisms (Figure 1).

#### 3.2.2 Sleep quality

A total of 44.9% of students reported that they sleep 7 or more hours per night, with 51.2% rating the quality of their sleep as “good” or “very good”. The mean PSQI score was 6.6 ± 3.2 points. Furthermore, 70.9% of the students scored more than 5 points (5+). The gender differences were not significant. Finnish students significantly revealed the best sleep quality, while students from Greece and Portugal the lowest (Table 3). Additionally, 13.2% of students admit taking sleeping pills. 50.3% of all students experience daytime tiredness at least 3 times a week. Meanwhile, 19.7% of students (women significantly more frequently) mention concentration difficulties, and 16.2% claim a lack of energy.

Significant differences between countries were found for fatigue (*p* = 0.001), concentration problems (*p* = 0.007), and lack of energy (*p* < 0.001). Students from Kosovo seem to suffer the least concentration problems (5%), whilst displaying the highest energy levels (45%). Conversely, Italian students reported the highest incidence of concentration problems (25%) and lack of energy (25%).

PSQI score significantly correlates with USOS score (*r* = 0.39, *p* < 0.001), with no significant differences by gender or country. Moreover, a strong positive correlation between concentration problems and the lack of energy (*r* = 0.44, *p* < 0.001), as well as between the lack of energy and fatigue (*r* = 0.47, *p* < 0.001); concentration problems and fatigue (*r* = 0.29, *p* < 0.001); and PSQI scores and fatigue (*r* = 0.34, *p* < 0.001) were confirmed, regardless of gender, country, or university.

#### 3.2.3 Dietary habits

In total, 71.5% of students comply with the recommendation of consumption of at least 1.5 L of water/day (34), men significantly more often than women (84% vs. 66%; *p* < 0.001). Water drinking significantly (*p* = 0.003) differs between countries: students from Portugal consumed less (54%), on the contrary students from Kosovo (86%) significantly more often meet the minimum of 1.5 litters of water/day.

The recommended amount (at least 2 or more) of servings of fruit per day were consumed on average by 31.8% of students (33% of women vs. 30% of men). Students from Spain (71%) and Italy (50%) eat significantly (*p* = 0.001) more fruit than students from Greece or Slovakia (22%). Three or more portions of vegetables per day were consumed by only 14.4% students (16% of women vs. 11% of men), the most by Italian and Spanish students (31 and 29%) and the least students from Kosovo (9%).

A positive correlation between BMI and fruit and vegetable intake was statistically significant. Surprisingly, 43% of obese students consumed 2 or more servings of fruit per day, compared to only 31% of students with a normal BMI. Similarly, the consumption of 3 or more servings of vegetables per day was consumed by 30% of obese vs. 13% of students with a normal BMI.

A third of students claimed that they do not consume alcohol (33.2%), of the remaining most students consume less than 3 drinks (44.5%) per week. Men consume significantly more drinks per week than women (*p* = 0.028). Significantly more students from Kosovo do not consume any alcohol (77%; 17; *p* < 0.001). In contrast, most students consuming one or more drinks/week are from Italy (82%; 13).

The education in the field of nutrition is considered important by 97.2% (1,045) of students. However, they evaluate its quality rather negatively on average (−0.44 points on −2 to +2 scale, SD 1.94). Only 19% (204) of students do not see any problem in nutrition education at their school. For 34.5% of students, both the quality and quantity of the education they receive is insufficient, while for 21.1% only quality and for 22.9% only quantity is insufficient.

#### 3.2.4 Physical activity

On average, students reported 1,891 ± 1,458 METs-min/week based on IPAQ (vigorous, moderate, and walking). It means that only 30.5% reached the recommended level of health enhancing physical activity (HEPA), moreover 16.1% were classified as “inactive”, even though 96.7% of students believed that physical activity affected their mental health. Men were significantly more active and classified as HEPA than women (2,107 ± 1,522 METs-min/week; vs. 1,782 ± 1,414 METs-min/week, *p* < 0.001). The highest rate of inactivity was found in Portuguese students (39%) and the lowest in the Czech Republic (8%). Conversely, the most active (HEPA category) students were found in Finland (41%), but none of the students at the University of Oulu met the HEPA.

Contrary to WHO recommendation, only 26% of students met 150+ min/week of moderate physical activity, and only 8% met more than 300 min/week. The150 min threshold for moderate activity and walking (combined) was met by 78.9% of students, and over 300 min by 51%. About 47% of students met 75 min/week of vigorous activity, with 29% reaching 150+ min/week. A total of 67% of students did strengthening exercises 2 or more times a week (Table 3).

Higher physical activity (METs-min/week) was associated with better quality of life (USOS score) (*r* = −0.19, *p* < 0.001) in men. Increasing the amount of METs-min/week had a significant effect on WHOQOL-BREF (*r* = −0.1, *p* < 0.001) and psychological health (*r* = −0.1, *p* < 0.001) in men.

#### 3.2.5 Paid employment

A third (33.4%) of students, regardless of gender, have paid employment (on average 4.6 ± 8.0 h per week), 40.1% of them work in their field of study. Significant differences were found between countries (*p* < 0.001). The largest number of students work at Slovak universities (58%), the least in Portugal (16%), and Spain (17%). No relationship between time spent at work and quality of life (USOS) was found.

#### 3.2.6 University background and study conditions

**Figure 2:**
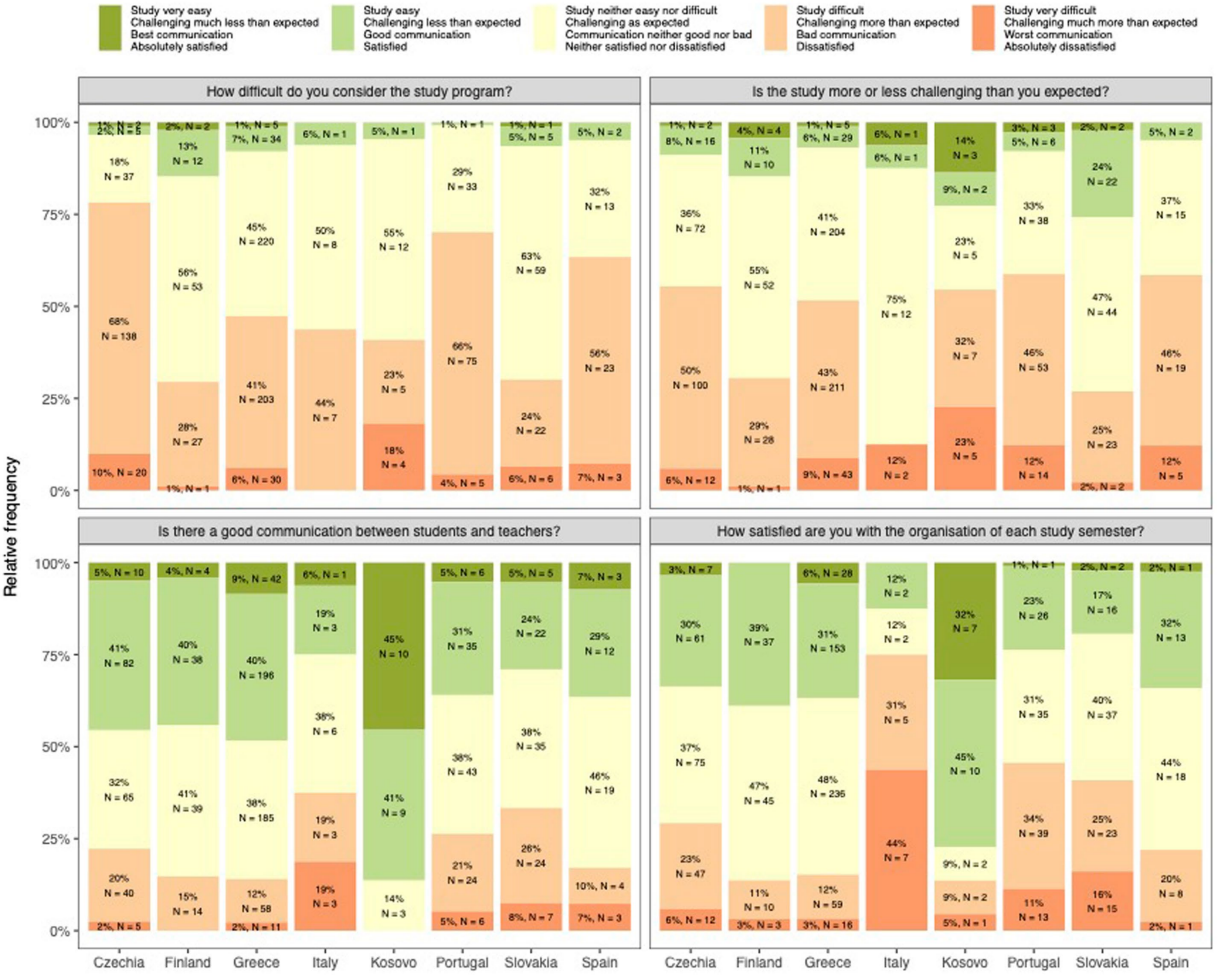
Summary of students satisfaction with study programme.

Students’ assessment of their studies is exactly in line with their expectations (average 0, maximum −2/+2). They are neither satisfied nor dissatisfied with the communication with teachers and the organisation of the study programme. There were no gender differences (*p* > 0.05). However, students differed between countries in all four aspects (*p* < 0.001, Figure 2).

Students from the Czech Republic, Portugal, and Spain found studying more difficult than other students. Students from the Czech Republic, Greece, Kosovo, Portugal, and Spain found studying more challenging. Students from Kosovo significantly reported the best communication and study organisation and were most satisfied with the materials provided (*p* < 0.001, Figure 2). Students from Italy reported the worst communication, organisation and inadequate materials provided (in the latter case together with Slovak students). Students for whom studying is more difficult than they expected also showed higher USOS scores (*p* < 0.001 and *r* = 0.22), more so for men (*r* = 0.31 vs. *r* = 0.15).

Overall, 11% considered the information received during lessons sufficient to pass the exam without studying external materials, while 48.1% must study from external materials received from teachers and even 32.2% must find external materials independently. Only 8.5% felt that there was not enough information for their exam.

**Figure 3:**
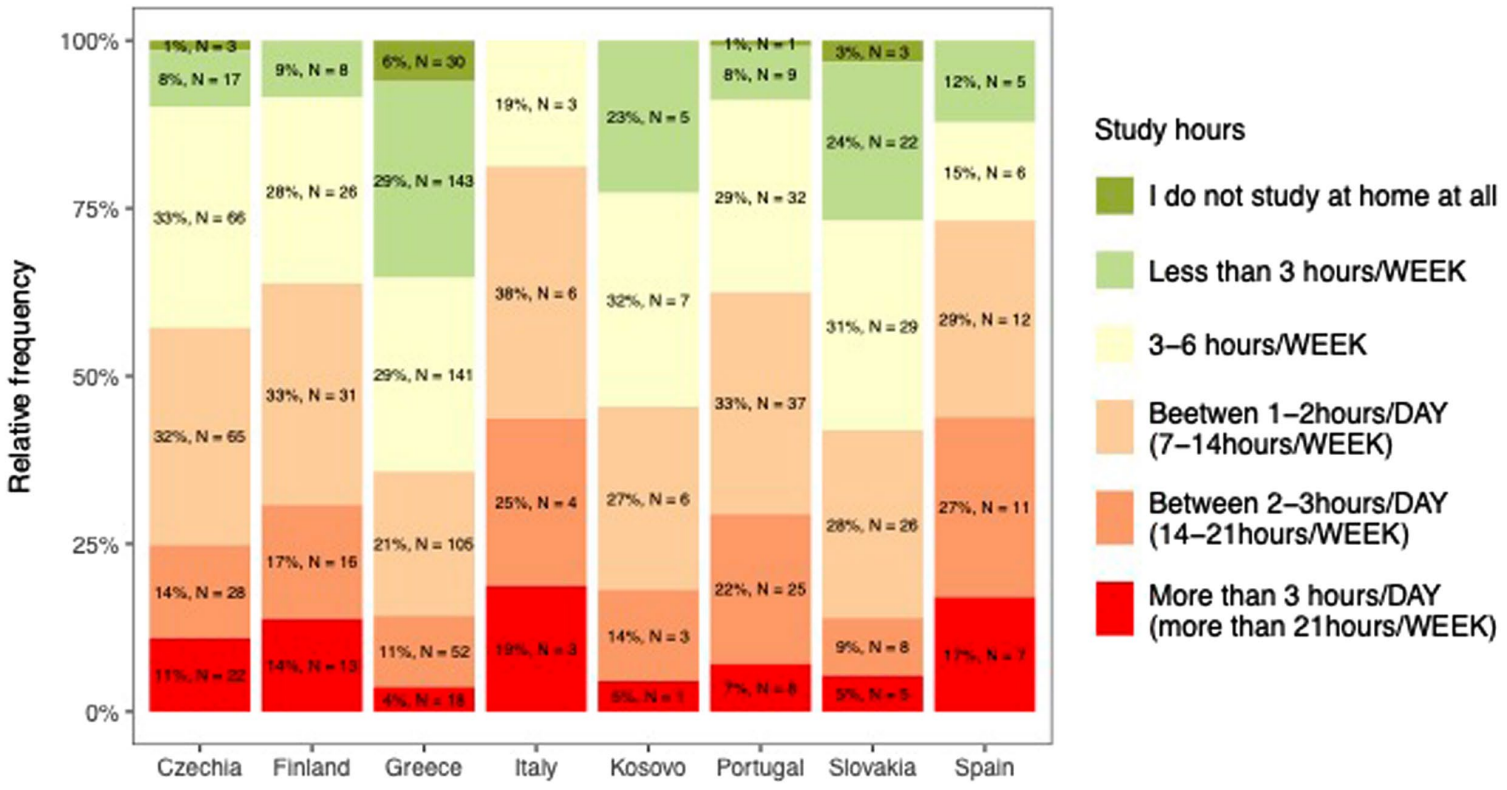
Amount of study hours among students.

On average, students’ study 8.6 ± 6.0 h per week, with women studying significantly more (9 ± 6.1 h vs. 7.9 ± 5.9, *p* = 0.004). Only 3.5% of students do not study at home at all, 19.6% study less than 3 h/week, 29% study 3–6 h/week, 27% study 1–2 h/day, 13.8% study 2–3 h/day and 7.2% study more than 3 h/day. Men study significantly less at home and if they do, they study less than 3 h/day. Italian students study significantly the most (Figure 3), while students from Greece and Slovakia study the least (*p* < 0.001). Those who spend more time studying also report that their studies are more demanding than they expected (*p* < 0.001 and *r* = 0.1). No association was found between the number of study hours and stress levels (USOS questionnaire, *p* = 0.87), sleep quality (PSQI, *p* = 0.42) or physical activity (METs *r*, *p* = 0.32).

## 4 Discussion

### 4.1 Stress and mental health

Although most of the students in this study are satisfied with their mental health, 21.5% perceive it as neutral to poor (WHOQOL-BREF) and even 65.6% show higher levels of distress (USOS). It is in accordance with findings in medical students (17), students of social and health sciences (35), and students of physiotherapy and dentistry (36).

Our work and the previous studies (2, 4, 16, 37–40) suggest differences in levels of stress and quality of mental health, with women generally more vulnerable, although one study reported no relationship between stress and gender (41). The most stressful are academic factors (2, 3, 16, 38, 42) such as the amount of material students must learn, the overall stress load at school, the “vastness” of the school curriculum and frequent tests (43). Our students also showed an association between higher levels of stress and subjective quality of life, as did the review (44).

### 4.2 Sleep quality

Sleep problems were reported by 70.5% of our students, aligning with findings in previous studies (24, 25, 27, 45–47). However, this contrasts with results from studies (19, 48, 49) that did not utilize the PSQI. Notably, while sleep problems are more frequently reported by women in some studies (19, 46), this trend was not observed in our study.

A total of 45% of our students sleep the recommended number of hours (more than 7), which is a lower percentage than in study (25) which indicates 64.8%, and higher than in authors (24) who reported only 20% (50, 51). In our case, 13.2% of students take sleep medication, mostly less than once a week. It is a higher percentage in comparison to other studies that indicate its use in 9–10% (25, 48) or only 6–4% (47, 49).

Our work confirmed relationship between the quality of sleep (PSQI) and the degree of perceived stress (USOS), similarly to studies (27, 52, 53); as well as between PSQI and quality of life, similarly to authors (8, 44, 47).

### 4.3 Dietary habits

Nutrition plays a crucial role in maintaining good health and preventing chronic diseases (54). However, studies show that many students in health professions do not follow dietary recommendations, putting themselves at risk of disease.

Our students did not meet the recommendations in consuming the recommended amount of liquids, fruit, and vegetables. From this point of view, they had a worse quality of diet on average, other studies showed the same result (45, 55, 56).

A total of 44.5% of students in our study consume alcohol less than three times a week, 22.3% consume three or more drinks a week. In a study of Hungarian university students, 35.4% of students consumed one drink per week, and students who consumed 3–7 drinks (10%) consumed them mostly at one time (57).

In our case, 97% of students believe that education in the field of nutrition is important, the same result we can find in (58), where 92% of students think this, and in their case, 30% of respondents consider current education in the field of nutrition as sufficient, in our case this is shared by 21.5% of students, the rest are dissatisfied with the quality and/or quantity of education in nutrition. It is crucial for future health professionals to be well-versed in nutrition, however the results are unsatisfactory and do not show any particular healthy lifestyle of these students (21, 55).

### 4.4 Physical activity

Surprisingly, our expectation that students of physiotherapy are more active than is recommended by WHO (1,200–3,000 METs-min/week) was not confirmed. On the other hand, they are in accordance with these recommendations, similarly to previous studies (18, 59).

If we consider the question whether students meet the recommended level of health enhancing physical activity (HEPA) by the IPAQ score, only 30.5% met the level, similarly to authors (60). On the other hand, our students are more active than in studies (18, 61), where only 16–20% of highly active students are found, and in study (62) where, similarly to our study, only 16% of them are inactive.

The relationship between good mental health/lower stress and higher physical activity has been confirmed by our as well as other studies (26, 63, 64). In our case, the relationship between (1) USOS/physical activity and (2) subjective assessment of mental health/physical activity in men was confirmed. No association was found between PSQI and physical activity, which only adds to the confusion about the association between the two categories (65).

### 4.5 University background and study conditions

Universities offer great access to information and knowledge, teach how to study, acquaint students with the social reality, show different perspectives of the present society and culture, and allow the possibility to discuss serious issues and their social repercussions (66). On the other hand, universities can be a source of stress caused by academic obligations and constant assessment both by teachers and by students themselves.

Our study confirmed that each university has different conditions and offers different support (or it may be perceived differently by students), which can be reflected in the level of stress perception. We were surprised that students from Kosovo have the best perception of their university, which we explain by the fact that in the context of war they perceive everything more positively. The most dissatisfied students are from Italy and Slovakia.

## 5 Conclusion to discussion

In line with other studies, students in this study suffered from (1) higher stress levels (17, 35, 36, 43), with women being more susceptible (2, 4, 16, 38–40), (2) sleep problems (24, 25, 27, 45–47) that were not related to gender, which is in contrast to some studies (19, 48, 49), (3) poor dietary habits (45, 46, 56), and (4) met WHO recommendations for physical activity of 1,200–3,000 METs-min/week (18, 59) but only 30.5% were in compliance with health-enhancing physical activity (HEPA) (18, 61). Academic factors were the most stressful (2, 3, 42, 43). Stress, as in review (44), and quality of sleep, as in studies (8, 44, 47), was associated with subjective assessment of quality of life. An association between physical activity and sleep quality was found, contrary to studies (65). Almost all students (97%) thought that nutrition education was important but not sufficient (58). For more detailed results see Table 4.

**Table 4:** Comparison with other studies results.

Authors	Year	Participants	Results
Wassif et al.	2019	390 medical students, all years	66.1% of students reported higher stress levels
Aslan et al.	2020	358 social and health science students from 14 universities	71% of students reported higher level of perceived stress, 52% presented anxiety symptoms and 62% depression symptoms
Owczarek et al.	2020	105 physiotherapy and dentistry students	Mean results in both groups indicated a high level of perceived stress in both groups
Tucker et al.	2006	434 physiotherapy students	Female students reported higher academic stress than male, academic factors were the most stressful
Hodselmans et al.	2018	116 physiotherapy students	Female students were more vulnerable to stress
Moutinho et al.	2017	761 medical students	47.1% of students reported stress symptoms, women were more vulnerable to stress
Eller et al.	2006	413 medical students	21.9% of students reported anxiety symptoms, 30.6% reported depression symptoms – both higher in females
Volken et al.	2021	3,571 students +2,328 swiss national population	Female students had higher prevalence of depressive symptoms, than matching female population
Pacheco et al.	2017	Meta-analysis of 59 studies (on medical students)	Female gender was significantly associated with depression, anxiety and stress
Cetinkaya et al.	2022	219 nursing students	Female students reported higher anxiety scale scores
Akgun et al.	2003	141 university students	No relationship found between stress and gender
Jacob et al.	2013	312 physiotherapy, communication disorder and nutrition sciences students	Academic factors were the most stressful, perceived stress correlated with grading stress factors
Lavoie-Tremblay et al.	2022	26 nursing students	Academic sources of stress were the most stressful
Ghrouz et al.	2019	617 college students	30% of students reported anxiety and 18% depression 51% reported low physical levels, 51% poor sleep quality, correlation between higher physical activity and lower anxiety and depression was found. Poor quality was significantly positively associated with anxiety and depression
Chowdhury et al.	2017	460 university students	46.3% of students reported higher stress levels, most stressful were academic factors (vastness of the school curriculum and frequent tests)
Ribeiro et al.	2018	Review	Found association between higher levels of stress and subjective QoL, connection found between PSQI and QoL
Pagnin et al.	2014	127 medical students	65% of students reported sleeping problems, only 20% of students slept 7+ hours/day
Džaferović et al.	2023	125 medical students	75.8% of students suffered from poor sleep quality, 64.8% of students slept more than 7 h, 10.4% of students used sleep medication
Carpi et al.	2022	1,279 university students	65% of students reported poor sleep quality
Rafidah et al.	2009	141 technology university students	Students reported sleeping problems, bad quality of diet
Sk et al.	2017	576 medical students	70.4% of students reported sleeping problems (more in female)
Preišegolavičiūtė et al.	2010	450 medical, law, business and economy students	59.4% of students reported sleeping problems (more in female), 5.9% used sleeping medication, connection between QoL and quality of sleep was found
Rathi et al.	2018	166 university students	Only 32.5% of students reported sleeping problems, poor sleep was more frequent among females
Corrêa et al.	2017	450 medical students	Only 39.5% of students reported sleeping problems, 8.6% used sleep medications
Zailinawati et al.	2009	555 medical students	Only 16.1% of students reported bad sleep quality, 3.9% used sleeping medication
Taylor et al.	2013	1,074 college students	Connection between worse quality of sleep and higher reported stress was found
Alyoubi et al.	2021	582 university students	Higher level of insomnia was associated with higher levels of stress
Ramón-Arbués et al.	2022	868 university students	Higher satisfaction with sleep and diet quality were associated with higher QoL
Bernal-Orozco et al.	2020	276 medical, nutrition and dentistry	Students reported poor quality of diet
Hilger et al.	2017	689 university students	Students reported poor quality of diet
Breitenbach et al.	2016	5,174 university students	35.4% of students consumed one alcoholic drink/week
Mogre et al.	2018	207 medical students	Education in nutrition is important for 92% of students, 70% of theme were dissatisfied with their education
Szypowska et al.	2020	165 cosmetology and physiotherapy students	Students reported poor quality of diet
Ranasinghe et al.	2018	115 physiotherapy students	Only 16% of students were HEPA and 48.7% were inactive
Rodríguez-Larrad et al.	2021	13,756 university students	Students are in accordance with WHO recommendations to 1,200–3,000 METS-min/week
Kgokong et al.	2020	296 physiotherapy students	Only 37.5% of students engaged in high physical activity
Zalewska et al.	2021	141 physiotherapy students	Only 19.9% of students fulfilled HEPA, and 40.4% had low physical activity, more physical activity had positive effect on mental health
Dąbrowska-Galas	2021	308 medical students	Only 19% of students were inactive
Kowalska et al.	2021	110 physiotherapy students	Relationship between good mental health/lower stress and higher physical activity has been confirmed
Chew et al.	2019	633 medical students	For 94.8% of students’ physical activity can lead to preventing diseases and to 70.9% it can treat diseases
Pacheco Salles et al.	2022	218 physiotherapy students	Relationship between good mental health/lower stress and higher physical activity has been confirmed
Memon et al.	2021	Meta-analysis of 29 studies	No connection between physical activity and quality of sleep

### 5.1 Limitations

The inhomogeneous distribution of students within universities and countries is the main limitation of the study. Some faculties obtained a very small sample of students, which can significantly distort the results of the study. In addition, in the first place, the timing of the data collection was not planned to coincide with the transition between two semesters and two academic years. However, some universities did not manage to obtain the necessary permissions from the ethics commission, or they did not manage to organise the data collection before the start of the summer examination period, and thus the data collection was extended into the winter semester. This is a reason why differences between semesters were not included in the analysis, although this was the primary intention. Also, the English in which the questionnaires were written may have limited some students from participating. As well as language, the length of the questionnaire could affect the response rate, as it took approximately half an hour to complete.

Differences in COVID restrictions between countries and institutions during the pandemics may have influenced the results. This issue was not specifically addressed in the questionnaire since the data were collected after the acute phase of COVID pandemic and the involved countries no longer had any specific restrictions on physical presence in the classroom. It is true that the previous different effects of COVID-related in the various countries may have influenced students’ attitudes. Nevertheless, we consider the study a success given its important information on health-related quality of life in physiotherapy students over a large number of countries.

## 6 Conclusion

In this study, we demonstrated that physiotherapy students, whose future profession requires good physical condition, are burdened with stress, and suffer from sleep disorders. Although they are educated in the field focused on the deterioration of health, they do not follow the recommendations regarding nutrition. Although the emphasis of their education is focused on physical fitness and quality of movement, their own physical activity is sometimes insufficient.

Further, there are significant differences in experienced stress, subjective assessment of mental health, quality of sleep, dietary habits, and amount of physical activity between universities. It would be advisable to take an example from universities that offer study conditions that students perceive as comfortable, and therefore prepare them well for their profession.

## Data availability statement

The raw data supporting the conclusions of this article will be made available by the authors, without undue reservation.

## Ethics statement

The studies involving humans were approved by Ethic Committee, Charles University, Third Medical Faculty, Ruská 87, Praha 10, 100 00. The studies were conducted in accordance with the local legislation and institutional requirements. The ethics committee/institutional review board waived the requirement of written informed consent for participation from the participants or the participants’ legal guardians/next of kin because the study was an online questionnaire that was distributed via online link and before starting to fill in the questionnaire, the students agreed that the provided data will be used for analytical processing.

## Author contributions

MS: Conceptualization, Investigation, Methodology, Project administration, Writing – original draft. KŘ: Conceptualization, Funding acquisition, Investigation, Methodology, Project administration, Writing – original draft, Writing – review & editing. JJ: Investigation, Methodology, Writing – original draft, Writing – review & editing. MP: Data curation, Formal analysis, Investigation, Methodology, Writing – original draft, Writing – review & editing. JR: Investigation, Methodology, Writing – original draft, Writing – review & editing. MÄ: Investigation, Methodology, Writing – original draft, Writing – review & editing. MŠ: Investigation, Writing – review & editing. AK: Investigation, Writing – review & editing. EŽ: Investigation, Writing – review & editing. TK: Investigation, Writing – review & editing. DP: Investigation, Writing – review & editing. BB-G: Investigation, Writing – review & editing. DB: Investigation, Writing – review & editing. EB: Investigation, Writing – review & editing. PG: Investigation, Writing – review & editing. JA-G: Investigation, Writing – review & editing. NS: Investigation, Writing – review & editing. AP: Investigation, Writing – review & editing. MK-Y: Investigation, Writing – review & editing. M-LL: Investigation, Writing – review & editing. JK: Investigation, Writing – review & editing. GB: Investigation, Writing – review & editing. MH: Investigation, Writing – review & editing. AA: Investigation, Writing – review & editing. SC-A: Investigation, Writing – review & editing.
