# Corrigendum: Quality of life and quality of education among physiotherapy students in Europe

**DOI:** 10.3389/fmed.2024.1408214

**Published:** 2024-04-08

**Authors:** Michaela Schramlová, Kamila Řasová, Johanna Jonsdottir, Markéta Pavlíková, Jolana Rambousková, Marja Äijö, Martina Šlachtová, Alena Kobesová, Elena Žiaková, Turhan Kahraman, Dagmar Pavlů, Beatriz María Bermejo-Gil, Daphne Bakalidou, Evdokia Billis, Papagiannis Georgios, José Alves-Guerreiro, Nikolaos Strimpakos, Aleš Příhoda, Marika Kiviluoma-Ylitalo, Marja-Leena Lähteenmäki, Jana Koišová, Gentiana Berisha, Magdalena Hagovská, Anna Laura Arca, Sara Cortés-Amaro

**Affiliations:** ^1^Department of Rehabilitation, Third Faculty of Medicine, Charles University, Prague, Czechia; ^2^IRCCS Fondazione Don Carlo Gnocchi ONLUS, Milan, Italy; ^3^Department of Hygiene, Third Faculty of Medicine, Charles University, Prague, Czechia; ^4^Savonia University of Applied Sciences School of Health Care, Kuopio, Finland; ^5^Department of Physiotherapy, Faculty of Physical Culture, Palacky University, Olomouc, Czechia; ^6^Department of Rehabilitation and Sports Medicine, Second Faculty of Medicine, Charles University and University Hospital Motol, Prague, Czechia; ^7^Department of Physiotherapy, Faculty Nursing and Professional Health Studies, Slovak Medical University in Bratislava, Bratislava, Slovakia; ^8^Department of Health Professions, Faculty of Health and Education, Manchester Metropolitan University, Manchester, United Kingdom; ^9^Faculty of Physical Education and Sport, Charles University, Prague, Czechia; ^10^Department of Nursery and Physiotherapy, Faculty of Nursery and Physiotherapy, Universidad de Salamanca, Salamanca, Spain; ^11^Laboratory of Neuromuscular and Cardiovascular Study of Motion (Lanecasm), Department of Physiotherapy, University of West Attica, Egaleo, Greece; ^12^Department of Physiotherapy School of Health Rehabilitation Sciences, University of Patras, Aigio, Greece; ^13^Biomechanics Laboratory, Physiotherapy Department, University of the Peloponnese, Sparta, Greece; ^14^Center for Innovative Care and Health Technology (ciTechCare), School of Health Sciences (ESSLei) Polytechnic of Leiria, Leiria, Portugal; ^15^Health Assessment and Quality of Life Lab Department of Physiotherapy, University of Thessaly, Volos, Greece; ^16^Division of Musculoskeletal & Dermatological Sciences, University of Manchester, Manchester, United Kingdom; ^17^Department of Health Care Disciplines and Population Protection, Faculty of Biomedical Engineering, Czech Technical University in Prague, Prague, Czechia; ^18^SAMK – Satakunta University of Applied Sciences, Pori, Finland; ^19^Tampere University of Applied Sciences, Tampere, Finland; ^20^Faculty of Health Sciences, University of Ss. Cyril and Methodius in Trnava, Trnava, Slovakia; ^21^Universum International College Pristina, Pristina, Kosovo; ^22^Department of Physiatry, Balneology, and Medical Rehabilitation, Faculty of Medicine, PJ Safarik University, Kosice, Slovakia; ^23^Coordinator of Physiotherapist School Traineeship AOU, Sassari, Italy; ^24^Physiotherapy in Motion, Multispecialty Research Group (PTinMOTION), Department of Physiotherapy, Faculty of Physiotherapy, University of Valencia Gascó Oliag n Valencia, Valencia, Spain

**Keywords:** students, physiotherapy, stress, nutrition, sleep, physical activity

In the published article, an author name was incorrectly written as [Sara Laura Cortés-Amaro]. The correct spelling is [Sara Cortés-Amaro].

The authors apologize for this error and state that this does not change the scientific conclusions of the article in any way. The original article has been updated.

