# Supplementary material for: Quality of life and quality of education among physiotherapy students in Europe
Source: Front Med (Lausanne). 2024 Feb 19;11:1344028. doi: 10.3389/fmed.2024.1344028 (PMC10936755; doi:10.3389/fmed.2024.1344028)
Supplement: Supplementary file 1 [file Data_Sheet_1.docx]

Supplementary Material

# Supplementary Data - Questionnaire for Students

Dear student,

thank you for participating in the questionnaire survey focused on the quality of life and quality of education of the students studying bachelor program of physiotherapy, and its comparison between different European universities.

The questionnaire is anonymous. It consists of **3 major domains** (general information about the respondent; quality of life and quality of education). The quality-of-life domain is divided into 5 subcategories (stress; sleep quality; nutritional habits; physical activity; paid job). The quality-of-education domain is divided into 2 subcategories (satisfaction with the study program and knowledge of the physiotherapy methods).

It takes about 20-30 minutes to fill in the questionnaire.

Please mark the appropriate response just by yourself. If you are not sure which answer to select, please choose the answer that comes closest to your opinion.

It is possible to mark more than one answer in some questions (= multiple choice), please, mark all the items describing your opinion.

Please answer all the compulsory questions. At the end of each domain, there is an **open question to add any thoughts or comments that come to your mind while filling the specific domain.** These questions are optional; however, your feedback might be useful for improving the questionnaire.

If you need help, please contact us by email **(michaelaschramlova@gmail.com).**

Thank you very much for your cooperation!

This statement describes how your personal information will be processed in the study: http://www.ceros.mobi/wp-content/uploads/2021/02/PRIVACY-STATEMENT.pdf.

A link for the agreement of the ethical committee from the Czech Republic:

http://www.ceros.mobi/wp-content/uploads/2021/02/souhlas-etick%C3%A9-komise.pdf

**By submitting, you agree with participating in the study, and all given data can be analysed and anonymously used.**

Online link: https://www.survio.com/survey/d/S9P9T7Q4H8E7A7R5I

***Part 1. - General Information***

- Gender
  - Female / Male / Different: specify.
- Age? – specify.
- Weight? – specify in kg.
- Height? – specify in cm.
- University? - Specify
- Current semester of study?
  - 1^st^, 2^nd^, 3^rd^, 4^th^, 5^th^, 6^th^, 7^th^, Different: specify.
- In your opinion, how good are you in English?
  - A1 – Beginner, A2 – Elementary, B1 – Intermediate, B2 – Vantage or Upper Intermediate, C1 – Advanced, C2 – Master of Proficiency

***Part 2. – Quality of Life***

#### Stress and Mental Health

- How would you rate your quality of life?
  - 1 - Very poor, 2 - poor, 3 - neither poor nor good, 4 - good, 5 - very good
- How satisfied are you with your mental health?
  - 1 - Very dissatisfied, 2 - dissatisfied, 3 - neither dissatisfied nor satisfied, 4 - satisfied, 5 - very satisfied
- How much do you enjoy life?
  - 1 - Not at all, 2 - a little, 3 - a moderate amount, 4 - very much, 5 - extremely
- To what extent do you feel your life to be meaningful?
  - 1 - Not at all, 2 - a little, 3 - a moderate amount, 4 - very much, 5 - extremely
- How satisfied are you with yourself?
  - 1 - Very dissatisfied, 2 - dissatisfied, 3 - neither dissatisfied nor satisfied, 4 - satisfied, 5 - very satisfied
- To what extent do you feel you have control over your life?
  - 1 - not at all, 2 - a little, 3 - a moderate amount, 4 - very much, 5 - extremely
- To what extent has each of the following been a source of stress to you in the last year (which of these following topics were stressful for you)?
  - Chose 1 - If you do not consider it stressful at all, 2 – a little stressful, 3 - a moderately stressful, 4 – a very stressful, 5 - an extremely stressful
  - ACADEMIC ISSUE
    - Amount of materials to be learnt in the course
    - Time demands of the course
    - Intellectual demands of the course
    - Physical demands of the course
    - The overall level of stress (at school)
    - Uncertainty (doubt) about the expectations in the course
- PERSONAL ISSUES
  - Stressful events (not at school but in your personal life)
  - Mood
  - Relation with family members
  - Relation with partner (wife/husband, girl/boyfriend…)
  - Physical health
  - Psychological health
  - Loneliness
- FINANCIAL ISSUES
  - Personal finances (food, personal things, clothes…)
  - Cost of books/equipment
  - Transportation
  - Accommodation
  - Do you have university fee?
    - - NO x YES
    - How stressful do you consider the university fee
    - 1 -not at all, 2- a little stressful, 3 – moderately stressful, 4 – very stressful, 5 – extremely stressful
- Which of these stress coping strategies, are you using the most often? (One answer)
  - I do not use any stress coping strategy
  - Meditation/mindfulness
  - Breathing methods
  - Relaxation techniques (e.g., yoga)
  - Walking (e.g., in the nature)
  - Sport activity (active sports, running, swimming etc.)
  - Socializing with friend and/or family, go to movies, theatre, concert etc.
  - Self-time in quiet spot (reading book, listening to music, just resting)
  - Different: specify
- Do you have any reminder for the part “Stress and mental health”? Anything that was not mentioned above, but you think is important? *(OPTIONAL QUESTION)*

#### Sleep Quality

Questions related to **usual** sleep habits**.**

- When have you usually gone to bed at night?
  - Before 9 pm, 9pm-10pm, 10-11pm, 11-12pm, 12pm-1am, 1-2am, after 2am
- How long (in minutes) has it usually takes you to fall asleep each night?
  - Less than 5min, 5-15min, 15-30min, 30min-1hour, more than 1 hour
- When have you usually gotten up in the morning?
  - Before 5am, 5-6am, 6-7am, 7-8am, 8-9am, 9-10am, after 10 am
- How many hours of actual sleep do you get at night? (may be different from hours, you spend in bed)
  - Less than 4 hours, 4-5hours, 5-6 hours, 6-7 hours, 7-8 hours, 8-9 hours, more than 9 hours
- How would you rate your sleep quality overall?
  - 1 - very poor, 2 - poor, 3 - neither poor nor good, 4 - good, 5 - very good
- How often have you had trouble sleeping because you… (1 -not during the past month, 2 - less than once a week, 3 - once or twice a week, 4 - three to five times a week, 5 – six to seven times a week)
  - Cannot get to sleep within 30 min
  - Wake up in the middle of the night or early morning and have difficulty to falling asleep
  - Must get up to use the bathroom
  - Cannot breathe comfortably
  - Caught or snore loudly
  - Feel too cold
  - Feel too hot
  - Had bad dreams
  - Have pain
- How often have you take the medicine (prescribed / “over the counter” to help you sleep?
  - 1 - no during the past month, 2 - less than once a week, 3 - once or twice a week, 4 - three to five times a week, 5 – six to seven times a week
- How often have you had trouble staying awake while driving, eating meals, engaging the social activity?
  - 1 - not during the past month, 2 - less than once a week, 3 - once or twice a week, 4 - three to five times a week, 5 – six to seven times a week
- Which of the following thing **most often** cause you stay up late into the night?
  - Studying/course assignments
  - Socializing with friends/family
  - Internet – social media
  - Video games/tv/computer playing
  - Different: specify
- How often did you feel tired during the past month?
  - 1 - not during the past month, 2 - less than once a week, 3 - once or twice a week, 4 -three to five times a week, 5 – six to seven times a week
- After sleep deprivation (condition of not having enough sleep) do you experience some physical issue(s) – like headache, nausea, vomiting etc.? *You can choose more options.*
  - Headache
  - Nausea
  - Vomiting
  - Tiredness – exhaustion
  - Deteriorated attention
  - Different: specify
  - I do not suffer from sleep deprivation
- How well are you able to concentrate?
  - 1 – not at all (have huge problem with concentration)
  - 2 – difficult concentration
  - 3 – neither difficult concentration nor good concentration
  - 4 – good concentration (sometimes have difficulty to concentrate)
  - 5 – great concentration, do not have any problem with concentration
- Do you feel you have enough energy for everyday life?
  - 1 – not at all
  - 2 – usually I am without energy
  - 3 – sometimes I feel without energy, sometimes I feel full of energy (50% of time I am full of energy, 50% I am without energy)
  - 4 – usually I feel full of energy
  - 5 – 100% of time I feel full of energy
- Do you have any remark for the part “Sleep quality”? Anything that was not mentioned above, but you think is important? *(OPTIONAL QUESTION)*

#### Physical Activity

- During the typical 7-Day period….
  - …how many days, do you exercise **for total of at least 30 minutes?** This includes things like jogging, playing soccer, doing fitness, dance, home exercising, walking, lifting heavy things etc. (every exercise you can imagine) **Do not count housework, walking from place to place.**
    - 0 days, 1 day, 2 days, 3 days, 4 days, 5 days, 6 days, 7 days
  - …how many times per week, do you do workouts to build and strengthen your muscles (at least for 10 minutes)?
    - 1 - 0 times, 2 - once a week, 3 -twice a week, 4 -3 times a week, 5 - 4 times a week, 6 -5 times a week, 7 -6 times a week, 8 -7 times a week, 9 - different: specify
    - On average, how long does your exercise take?
      - 1 – 10 minutes
      - 2 – 20 minutes
      - 3 – 30 minutes
      - 4 – 45 minutes
      - 5 – 60 minutes
      - 6 – 90 minutes
      - 7 – Different: specify
  - … how many times per week, do you do **vigorous exercise** (take hard physical effort and make you breathe much harder than normal, at least for 10 minutes) like running, jogging, hockey, football, soccer, basketball, rugby, beach volleyball, squash, basketball, judo, karate, tae kwon do, jujitsu, jumping rope, jumping jacks, rowing (or rowing machine, stair climber machine), roller skating, swimming, long distance bicycling (more than 10 mph or on steep uphill terrain or stationary bicycling – using vigorous effort), boxing, aerobic walking and racewalking (5mph or faster), mountain climbing, roller skating or in-line skating, aerobic dancing (or different dance type but energetically!), tennis – singles, skiing (vigorous effort!) ?
    - 1 - 0 times, 2 - once a week, 3 -twice a week, 4 -3 times a week, 5 -4 times a week, 6 -5 times a week, 7 -6 times a week, 8 -7 times a week, 9 - different: specify
    - On average, how long does your one exercise take?
      - 1 – 10 minutes
      - 2 – 20 minutes
      - 3 – 30 minutes
      - 4 – 45 minutes
      - 5 – 60 minutes
      - 6 – 90 minutes
      - 7 – Different: specify
  - …. how many times per week, do you do **moderate exercise** (take moderate physical effort and make you breathe somewhat harder than normal, at least for 10 minutes) like fast walking (3-4,5 mph), easy hiking, roller skating (or in-line skating) at a leisurely pace, easy bicycling (5-9 mph, or stationary bicycling using moderate effort), easy swimming, water aerobics, yoga, ballroom dancing (or ballet, or different dance style with no maximal effort), alpine skiing, table tennis, tennis – doubles, golf, frisbee playing, archery, badminton, skiing with light effort, sailing, horseback riding etc.?
    - 1 - 0 times, 2 - once a week, 3 -twice a week, 4 -3 times a week, 5 -4 times a week, 6 -5 times a week, 7 -6 times a week, 8 -7 times a week, 9 – different: specify
    - On average, how long does your one exercise take?
      - 1 – 10 minutes
      - 2 – 20 minutes
      - 3 – 30 minutes
      - 4 – 45 minutes
      - 5 – 60 minutes
      - 6 – 90 minutes
      - 7 – Different: specify
  - …how many times per week do you do **meditation/relaxing exercise**? (yoga, tai-chi etc., at least for 10 minutes)
    - 1 - 0 times, 2 - once a week, 3 -twice a week, 4 -3 times a week, 5 -4 times a week, 6 -5 times a week, 7 -6 times a week, 8 -7 times a week, 9 – different: specify
    - On average, how long does your one exercise take?
      - 1 – 10 minutes
      - 2 – 20 minutes
      - 3 – 30 minutes
      - 4 – 45 minutes
      - 5 – 60 minutes
      - 6 – 90 minutes
      - 7 – Different: specify
  - …how many **DAYS** **per week** do you spend walking for at **least 10 minutes**? This includes at work and at home, walking to travel from place to place, and any other walking that you have done solely for recreation, sport, exercise, or leisure.
    - 1 - 0 days, 2 – 1 day a week, 3 -2 days a week, 4 -3 days a week, 5 -4 days a week, 6 -5 days a week, 7 -6 days a week, 8 -7 days a week, 9 – different: specify
    - On average, how much time do you usually spend walking **in total on one of those day**?
      - 1 – 10 minutes
      - 2 – 20 minutes
      - 3 – 30 minutes
      - 4 – 45 minutes
      - 5 – 60 minutes
      - 6 – 90 minutes
      - 7 – Different: specify
- How would you describe your physical activity in comparison with your peers?
  - 1 – my physical activity is MUCH WORSE in comparison with my peers
  - 2 – my physical activity is WORSE in comparison with my peers
  - 3 – my physical activity is SAME in comparison with my peers
  - 4 – my physical activity is BETTER in comparison with my peers
  - 5 – my physical activity is MUCH BETTER in comparison with my peers
- On average, how many steps do you take per day?
  - 1 - 0 – 2 500 steps per day
  - 2 – 2 500 – 5 000 steps per day
  - 3 – 5 000 – 7 000 steps per day
  - 4 – 7 000 – 10 000 steps per day
  - 5 – 10 000 – 15 000 steps per day
  - 6 – more than 15 000 steps per day
  - 7 – I don’t know
- Do you think that physical activity affects your mental health?
  - YES x NO
- Do you have any remarks for the part “Physical activity”? Anything that was not mentioned above, but you think is important? *(OPTIONAL QUESTION)*

#### Paid Job

- Do you currently have a paid job?  *-* YES x NO
- How many hours per week do you spend in your employment?
  - 1 – 5 hours or less per week
  - 2 - 6 to 10 hours per week
  - 3 – 11 – 15 hours per week
  - 4 – 16 - 20 hours per week
  - 5 – 21 - 25 hours per week
  - 6 - more than 26 hours per week
- Do you work in you study field? Wellness, fitness, trainer, retirement homes, hospitals etc.? – YES x NO
- Do you have any remarks for the part “Paid job”? Anything that was not mentioned above, but you think is important? *(OPTIONAL QUESTION)*

#### Nutritional Habits

- Do you drink at least 1.5 litres of drinks (water, tea, mineral water etc.) every day (Monday to Sunday)? YES x NO
- How many portion(s) of fruit do you approximately consume **per day**? (1 portion = 1x banana or 1x small apple or 1x cup of 100% fruit juice or 8 strawberries or 3 plums)
  - 1 – I do not eat fruit at all
  - 2 – 1 portion per day
  - 3 – 2 portions per day
  - 4 – 3 portions per day
  - 5 – 4 or more portions per day
  - 6- Different: specify
- How many portion(s) of vegetables do you approximately consume **per day**? (1 portions = 1x cup of 100% vegetable juice, 1x big tomato, 2x carrots, 1x pepper, 2x cups of fresh leaf vegetables (e.g., spinach, lamb’s lettuce etc.)
  - 1 – I do not eat vegetable at all
  - 2 – 1 portion per day
  - 3 – 2 portions per day
  - 4 – 3 portions per day
  - 5 – 4 or more portions per day
  - 6- Different: specify
- How many portions of legumes (peas, lentils, bean) do you consume **per week**?
  - 1 – I do not eat legumes at all
  - 2 – 1 portion per week
  - 3 – 2 portions per week
  - 4 –3 portions per week
  - 5 –4 or more portions per week
  - 6 - Different: specify
- How many portions of fresh, frozen, or canned fish do you consume **per week**? (1 portion = approximately 150g, normal can have usually 120-150g)
  - 1 – I do not eat fish at all
  - 2 – 1 portion per week
  - 3 – 2 portions per week
  - 4 –3 portions per week
  - 5 –4 or more portions per week
  - 6 - Different: specify
- Excluding the fish, how many times per WEEK do you consume meat? (1 portion = approximately 120-150g).
  - 1 – I do not eat meat at all
  - 2 – 1 portion per week
  - 3 – 2 portions per week
  - 4 –3 portions per week
  - 5 –4 or more portions per week
  - 6 - Different: specify
- How many sweetened beverages (“fizzy drinks”) do you consume per WEEK?
  - 1 – I do not drink sweetened beverages at all
  - 2 -1-2 drinks per week
  - 3 - 3-4 drinks per week,
  - 4 – 5-6 drinks per week
  - 5- 7 drinks per week (everyday)
  - 6 - several times a day = more than 7 times per week
  - 7 – different: specify
- Do you consume at least 30g of nuts (walnut, hazelnut, almond, peanut) or seeds (sunflower seed, linen seed…) or one-half of an avocado every day (Monday to Sunday)? (1-2 tablespoon(s) of sunflower seeds/day)
  - YES x NO
- How many times **per week** do you consume processed food (fried foods, sausage, packaged meals ready to heat and serve)?
  - 1 – I do not eat processed food at all
  - 2 – once a week
  - 3 – twice a week
  - 4 – 3 times per week
  - 5 – 4 times per week
  - 6 – 5 or more times per week
  - 7 – Different: specify
- How many times **per week do** you consume not prepared food at home?
  - 1 – I eat only food which I prepared at home
  - 2 – once a week
  - 3 – twice a week
  - 4 – 3 times per week
  - 5 – 4 times per week
  - 6 – 5 or more times per week
  - 7 – Different: specify
- How many times **per week** do you consume dessert food (e.g., cookies, crème caramel (flan), rice pudding, cakes) or sweets (candy, popsicles, chocolates)?
  - 1 – I do not eat dessert food or sweets at all
  - 2 – once a week
  - 3 – twice a week
  - 4 – 3 times per week
  - 5 – 4 times per week
  - 6 – 5 or more times per week
  - 7 – Different: specify
- How much alcohol do you consume approximately **per week**? (1 – standard alcoholic drink = 1 dcl of wine, 0,3 l of beer, 40 ml of distilled (e.g., vodka, rum, whiskey), 1,5 dcl of champagne)
  - 1 – I do not drink alcohol at all
  - 2 – 1-2 standard alcoholic drinks per week
  - 3 – 3-4 standard alcoholic drinks per week
  - 4 – 5-6 standard alcoholic drinks per week
  - 5 – 7 standard alcoholic drinks per week (everyday)
  - 6 – several drinks a day (more than 7 per week)
  - 7 – Different: specify
- What type of alcohol do you consume the most often?
  - beer, wine, champagne, distilled (vodka, rum, whiskey), different: specify
- How many cups of coffee/ or different caffeine sources (e.g.maté) do you approximately consume **per day**?
  - 1 - I do not drink coffee/different caffeine sources at all
  - 2 – 1 cup per day
  - 3 - 2 cups per day
  - 4 – 3 cups per day
  - 5 - 4 cups per day
  - 6 - 5 or more cups per day
  - 7 – different: specify
- Are you currently following a diet?
  - NO
  - Yes, as advised by my doctor for medical reason
  - Yes, it was my personal decision
- Do you currently smoke cigarettes, pipe, or other tobacco?
  - YES x NO
- Did you start smoke in the past?
  - YES – specify when did you stop
  - NO
- How would you describe your nutritional habits?
  - 1 -very poor, 2- poor, 3 -neither poor nor good, 4- good, 5 - very good
- How would you describe your health status in comparison to your peers?
  - 1 – MUCH WORSE than others
  - 2 - WORSE than others
  - 3 – SAME as others
  - 4 - BETTER than others
  - 5 – MUCH BETTER than others
- Do you think nutritional education is important for you as future physiotherapist?
  - YES x NO
- How satisfied are you with the level of education (quality and quantity) in nutrition at your university?
  - 1 - very dissatisfied
  - 2 - dissatisfied
  - 3 - neither dissatisfied nor satisfied
  - 4 - satisfied
  - 5 - very satisfied
- Please specify, where you see the biggest issues and what should be improved?
  - I do not see any problems.
  - The quantity in insufficient.
  - The quality is insufficient.
  - The quality and quantity are insufficient.
  - Different: specify
- Do you have any remarks for the part “Nutritional habits “? Anything that was not mentioned above, but you think is important? *(OPTIONAL QUESTION)*

***Part 3. – Quality of Education***

#### Study Programme, Study Environment

- How difficult do you consider the study program?
  - 1 - very easy, 2 - easy, 3 - neither easier nor difficult, 4 - difficult, 5 - very difficult
- Is the study more or less challenging than you expected?
  - 1- MUCH LESS challenging, 2 – LESS challenging, 3- SAME, 4 -MORE challenging, 5 -MUCH MORE challenging
- Do you think you get enough and complex information at your courses? *Please focused only on the special physiotherapy courses.*
  - YES I got enough information during the lecture, I do not need to study external materials to pass the exam
  - YES I got a lot of information, but I need to study independently external materials, but most of them I got from teacher
  - YES I got a lot of information, but I need to study independently external materials, which I need to search for first
  - NO, I do not get enough information and to pass the exam I must study independently external materials which I need to search for first
  - Different- specify
- Do you think there is a good communication between students and teachers? When we think about the exams, individual study issues (individual study programme etc)?
  - 1 – THE WORST communication
  - 2 – bad communication
  - 3- neither good nor bad communication
  - 4 – good communication
  - 5 – THE BEST communication
- How many hours do you spend studying?
  - 1 – I do not study at home at all
  - 2 - less than 3 hours/week
  - 3 - 3-6 hours/week
  - 4- between 1-2 hours/day (7-14hours/week)
  - 5- between 2-3hours/day (14 – 21 hours/week)
  - 6- more than 3hours/day (more than 21hours/week)
  - 7- different: specify
- How satisfied are you with the organisation of each study semester/year? (Having enough theoretical and practical/clinical teaching, etc.).
  - 1 – absolutely dissatisfied
  - 2 – dissatisfied
  - 3 – neither dissatisfied nor satisfied
  - 4 – satisfied
  - 5 – absolutely satisfied
- Please specify the biggest issue(s) you find in the field of organisation of the study year. (open question)
- What do you want to do after your bachelor’s degree graduation?
  - Work as physiotherapist. Bachelor’s degree is enough for me. Do not want to study anymore.
  - Work as personal trainee/ in fitness etc. but not as physiotherapist, but I will use knowledge gained during the study
  - Work in different field - not as physiotherapist, but do not want to study anymore
  - Will study for master’s degree in Physiotherapy
  - Want to study something different - no master’s degree in Physiotherapy
  - Different: specify
- Do you have any remarks for the part “Study programme, study environment”? Anything that was not mentioned above, but you think is important? *(OPTIONAL QUESTION)*

#### Knowledge of the Physiotherapy Methods and Approaches

- How well do you know the following methods and approaches?
- On a range between:
  - **1** - never heard of it
  - **2** - I have heard of it/or read about it but in a course, we have never been taught it
  - **3** - I have heard of it in a theoretical lesson, but we did not have any practical lesson
  - **4 -** did not have any theoretical lessons, but I was taught in practical seminars (e.g., in a hospital, etc.)
  - **5 -** I know the method theoretically and practically from lessons
- Aerobic training
- Aquatherapy
- Balance platform (proprioceptive posture therapy – 3D Spacecurl, PORTUROMED, vibration platform)
- Biofeedback (electrical stimulation, pulse magnetic field therapy, TENS)
- Bobath concept
- Breathing exercise – respiratory rehabilitation
- Brünnstorm approach
- Brunkow approach
- Brügger concept
- Constraint-induced movement therapy (CIMT)
- Cryotherapy
- Dual tasking (require performing two tasks simultaneously)
- Feldenkrais method
- Heat therapy (application of heat to the body for pain relief)
- Hippotherapy
- Nordic walking
- Manual therapy (joint mobilization/manipulation, kneading, manipulation of muscles)
- Music therapy
- Muscle stretching (improves muscle’s elasticity)
- Muscle strengthening (e.g., Resistant exercise to induce muscular contraction to build strength)
- Motor learning program
- Oriental methods (acupuncture)
- Pain control
- PNF – proprioceptive neuromuscular facilitation
- Perfetti approach
- Pelvic floor exercise
- Proprioneuro-physiological methods (post-isometric relaxation)
- Relaxation techniques
- Robotic-assisted rehabilitation therapy (e.g., Lokomat, Armeo)
- Sensory stimulation
- Simple devices (kinesio-tape, overball, mirror, thera-band, splints, standing frame)
- Transfer (training of transfers and ambulatory abilities)
- Vojta reflex locomotion
- Did you complete Sonography course (diagnostic use of ultrasound, E.g., for muscular rupture, inflammation etc.)?
  - 1 - never heard of it
  - 2 - I have heard of it/or read about it but in course we never been taught it
  - 3 - I have heard of it in theoretical lesson, but we did not have any practical lesson
  - 4 - did not have any theoretical lesson, but I was taught in practical seminars (e.g., in hospital etc.)
  - 5 **-** I know the method theoretically and practically from lessons
  - 6 – Different: specify
- If you completed sonography course (use of diagnostic ultrasound), was it a compulsory or voluntary subject?
  - I did not have it
  - Compulsory
  - Voluntary
- To what extend did you have a practical teaching of sonography during your whole study? (Use of diagnostic ultrasound e.g., to diagnose a rupture of the muscle, inflammation, or tendon structure changes etc.)
  - I did not have practical teaching of sonography
  - 1 – 5 hours
  - 6 – 15 hours
  - 16 – 30 hours
  - More than 31 hours
  - Different: specify
- Do you have any remarks for the part “Knowledge of physiotherapy methods”? E.g., are there any methods you can use /were taught on your university but were not mentioned above? *(OPTIONAL QUESTION)*
